# Low-molecular-weight heparins are superior to vitamin K antagonists for the long term treatment of venous thromboembolism in patients with cancer: a cochrane systematic review

**DOI:** 10.1186/1756-9966-27-21

**Published:** 2008-07-18

**Authors:** Elie A Akl, Maddalena Barba, Sandeep Rohilla, Irene Terrenato, Francesca Sperati, Paola Muti, Holger J Schünemann

**Affiliations:** 1 Department of Medicine, State University of New York at Buffalo, Buffalo, NY, USA; 2 Department of Epidemiology, Italian National Cancer Institute Regina Elena, Rome, Italy; 3 Department of Medicine, Maulana Azad Medical College, University Of Delhi, New Delhi, India

## Abstract

**Background:**

Cancer and its therapies increase the risk of venous thromboembolism. Compared to patients without cancer, patients with cancer anticoagulated for venous thromboembolism are more likely to develop recurrent thrombotic events and major bleeding. Addressing all important outcomes including harm is of great importance to make evidence based health care decisions. The objective of this study was to compare low molecular weight heparin (LMWH) and oral anticoagulants (vitamin K antagonist (VKA) and ximelagatran) for the long term treatment of venous thromboembolism in patients with cancer.

**Methods:**

A systematic review of the medical literature. We followed the Cochrane Collaboration methodology for conducting systematic reviews. We assessed methodological quality for each outcome by grading the quality of evidence using the Grading of Recommendations Assessment, Development and Evaluation (GRADE) methodology.

**Results:**

Eight randomized controlled trials (RCTs) were eligible and reported data for patients with cancer. The quality of evidence was low for death and moderate for recurrent venous thromboembolism. LMWH, compared to VKA provided no statistically significant survival benefit (Hazard ratio (HR) = 0.96; 95% CI 0.81 to 1.14) but a statistically significant reduction in venous thromboembolism (HR = 0.47; 95% (Confidence Interval (CI) = 0.32 to 0.71). There was no statistically significant difference between LMWH and VKA in bleeding outcomes (RR = 0.91; 95% CI = 0.64 to 1.31) or thrombocytopenia (RR = 1.02; 95% CI = 0.60 to 1.74).

**Conclusion:**

For the long term treatment of venous thromboembolism in patients with cancer, LMWH compared to VKA reduces venous thromboembolism but not death.

## Background

The presence of cancer increases the risk of venous thromboembolism four to six fold [1]. Cancer related interventions such as chemotherapy, hormonal therapy and indwelling central venous catheters also increase the risk of venous thromboembolism [1]. Similarly, patients undergoing surgery for cancer have a higher risk of venous thromboembolism than those undergoing surgery for benign diseases [2,3]. Furthermore, patients with cancer and venous thromboembolism have a higher risk of death than patients with cancer alone or with venous thromboembolism alone [4,5].

Cancer patients also have different benefits and risks from anticoagulant treatment than those without cancer. For instance, during oral anticoagulation therapy for venous thromboembolism, patients with cancer, compared to those without cancer, have higher incidence of recurrent venous thromboembolism (27.1 versus 9.0 events per 100 patient years, p = 0.003) and of major bleeding (13.3 versus 2.2 events per 100 patient years, p = 0.002) [6].

Three systematic reviews have compared low molecular weight heparin (LMWH) and vitamin K antagonists (VKA) in the long treatment of venous thromboembolism, but in populations not restricted to patients with cancer [7-9] The review by van der Heijden et al. did not complete a preplanned subgroup analysis in patients with cancer as the required data was not specifically reported [7] The review by Conti et al. did not conduct a meta-analysis in the subgroup of patients with cancer [8] In the review by Ioro et al. a meta-analysis in the subgroup of patients with cancer found no statistically significant difference in mortality (OR = 1.13; 95% CI 0.54, 2.38).

No systematic review has focused on the long term treatment of venous thromboembolism in patients with cancer. The above mentioned subgroup analysis did not report on the comparative safety of LMWH and VKA [9] The Cochrane Collaboration has recognized that addressing all important outcomes including harm is of great importance to make evidence based health care decisions [10]. In addition, an analysis that includes an evaluation of direct comparative trials and direct subgroup comparison could prevent the potential pitfalls of indirect subgroup analysis [11].

The objective of this study was to conduct a systematic review to compare the efficacy and safety of LMWH and oral anticoagulants for the long term treatment of venous thromboembolism in patients with cancer.

## Methods

### Eligibility criteria

We included RCTs including patients with cancer with a confirmed diagnosis of venous thromboembolism (deep venous thromboembolism (DVT) or pulmonary embolism). The venous thromboembolic event should have been diagnosed using an objective diagnostic test. RCTs should have compared long term treatment with LMWH versus oral anticoagulants (VKA or ximelagatran) and should have treated patient groups similarly apart from the intervention of interest.

### Outcomes of interest

Outcomes of interest included: survival, symptomatic recurrent DVT, symptomatic recurrent pulmonary embolism, major bleeding, minor bleeding, thrombocytopenia, and postphlebitic syndrome. We accepted the definitions of major bleeding, minor bleeding, thrombocytopenia and postphlebitic syndrome of the authors of the original studies as long as they were standardized.

### Data Sources and Searches

The search was part of a comprehensive search for studies of anticoagulation in patients with cancer. We electronically searched in January 2007 the following databases from the date of their inception: The Cochrane Central Register of Controlled Trials, MEDLINE, EMBASE and ISI the Web of Science (Additional file 1). We also hand searched the conference proceedings of the American Society of Clinical Oncology and of the American Society of Hematology. We reviewed the reference lists of included papers and used the related article feature in PubMed. We applied no language restrictions.

### Study Selection

Two reviewers independently screened the titles and abstracts for eligibility. We retrieved the full texts of articles judged as potentially eligible by at least one reviewer. Two reviewers then independently screened the full texts articles for eligibility and resolved their disagreements by discussion. We included studies published as abstracts only if authors supplied us with the necessary information about their methods and results.

### Data collection

Two reviewers independently extracted data using a standardized form and resolved their disagreements by discussion. Extracted data related to participant characteristics, the details of the interventions, the outcomes and methodological quality indicators. We contacted authors for incompletely reported data.

We assessed the following methodological criteria for each study: allocation concealment, blinding (patient, provider, outcome assessor, data analyst), whether the analysis followed the ITT principle, whether study was stopped early for benefit, and percentage of follow-up. We assessed the methodological quality for each outcome by grading the quality of evidence using the Grading of Recommendations Assessment, Development and Evaluation (GRADE) approach [12]. The GRADE approach involves making separate ratings for quality of evidence for each patient important outcome and identifies five factors that can lower the quality of the evidence when considering RCTs: study limitations relating to the above methodological criteria (lack of allocation concealment; lack of blinding; failure to adhere to an intention to treat analysis; stopping early for benefit; and large losses to follow-up), inconsistency of results, indirectness of evidence, imprecision, and publication bias [13].

We extracted time to event data by abstracting the log(hazard ratio) and its variance from trial reports; if these were not reported, we digitised the published Kaplan-Meier survival curves and estimated the log(hazard ratio) and its variance using Parmar's methods [14]. We also noted the minimum and maximum duration of follow-up, which are required to make these estimates. We performed these calculations in Stata 9, using a specially written program, which yielded the reported log(HR) and variance when used on the data presented in Table V of Parmar 1998 [14].

We also extracted categorical data necessary to conduct intention-to-treat analyses. We collected outcome event rates whenever they were reported in each trial. When the authors did not report and could not provide the number of events at specific time points, two biostatisticians estimated these numbers independently and in duplicate from survival curves, if available.

### Analysis

We calculated the agreement between the two reviewers for the assessment of trial eligibility using kappa statistic. We analyzed, when possible, both time to event data and binary data. For time to event data, we pooled the log(HR)s using a random-effects model and the generic inverse variance facility of RevMan 4.2. For binary data, for a specific outcome, and for each trial, we used the intention-to-treat principle to calculate the relative risk. We then pooled the results of trials with similar comparisons using a random-effects model.

We evaluated heterogeneity across trials using the I^2 ^statistics. I^2 ^describes the percentage of total variation across studies that is due to heterogeneity rather than chance [15]. The interpretation of I^2 ^depends on the magnitude and direction of effects as well as the strength of evidence for heterogeneity (e.g. P value from the chi-squared test, or a confidence interval for I^2^) [10]. We used the following classification based on the value of I^2 ^[15]: 0–30 = low; 30–60 = moderate and worthy of investigation; 60–90 = severe and worthy of understanding; 90–100 = allowing aggregation only with major caution.

We created inverted funnel plots of individual study results plotted against sample size in order to evaluate possible publication bias. We conducted sensitivity analysis by excluding the study of lowest methodological quality [16] and then a study that used a different initial anticoagulant in the two study arms (post hoc analysis) [17].

## Results

### Results of the search

**Figure 1:**
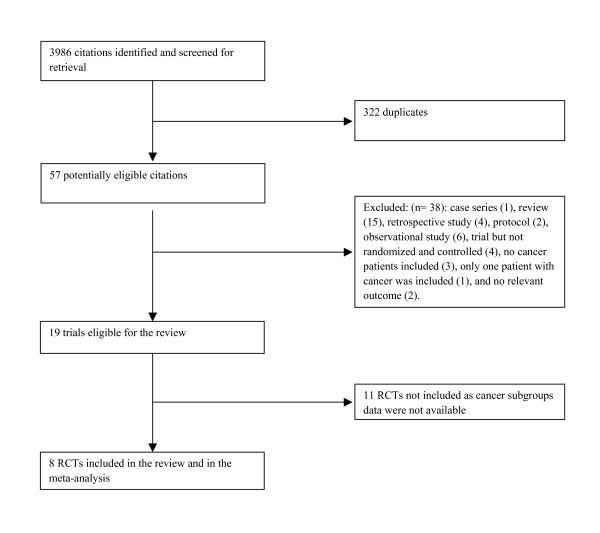
Trial flow in the systematic review of long term anticoagulation in patients with cancer and venous thromboembolism.

Figure 1 shows the trial flow. The search identified 3986 citations, including 322 duplicates. The title and abstract screening of the 3664 unique citations identified 57 as potentially eligible for this review. The full text screening excluded 40 citations for the following reasons: The reasons for excluding the 40 citations are as follows: case series (1), review (15), retrospective study (4), protocol (2), observational study (6), trial but not randomized and controlled (4), no cancer patients included (3), only one patient with cancer was included (1), and no relevant outcome (2), comparison of two LMWH (tinzaparin and dalteparin) (1), 18 months extended treatment with Ximelagatran versus placebo (1).

Of the remaining 17 eligible RCTs, 11 included patients with cancer as a subgroup the data of which was not reported and not obtainable from the authors [18-28]. We thus report data from eight RCTs, five published in full text [17,29-33] and one published as an abstract [16]. We also identified 2 publications related to RCTs we included in this review [34,35]. Agreement between reviewers for study eligibility was excellent (kappa = 0.94).

### Included studies

**Figure 2:**
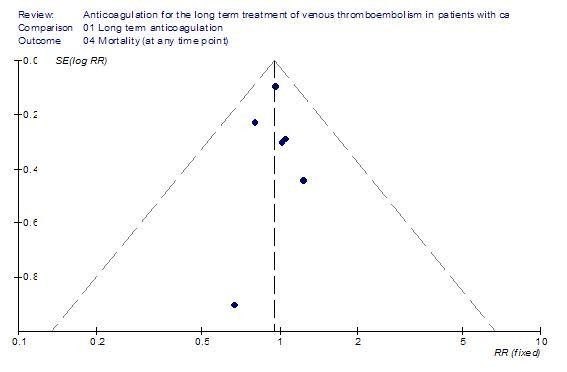
Inverted funnel plot for the mortality outcome in randomized controlled trials of long term anticoagulation in patients with cancer and venous thromboembolism.

Table 1 details the characteristics of the six included studies. Only one of these studies used a different initial anticoagulant in the two study arms (LMWH in the LMWH group and UFH in the vitamin K antagonist group) [17]. The inverted funnel plot for the outcome of all cause mortality did not suggest publication bias (Figure 2).

**Table 1:** Comparative table of randomized controlled trials comparing different types of anticoagulants for the long term treatment of venous thromboembolism in patients with cancer

**Study**	Methods*	**Interventions**†	**Participants**§	**Outcomes**§	**Notes**
López-Beret 2001	AC: Not clear Blinded: outcome assessors ITT analysis Sample size not calculated a priori 100% follow-up for primary outcome	Nadroparin 1.025 AXa IU/10 Kg twice daily for 3 days then randomized to Nadroparin 1.025 antiXa IU/10 Kg twice daily versus acenocoumarol (target INR 2–3) for 3–6 months. After the 3^rd ^month, nadroparin was switched to once daily. 68% of INR values were on target.	35 patients with known malignancy; treated for symptomatic DVT of the lower limbs; minimum age of 18	Death at 12 months	Funding: Not reported
Meyer 2002 (CANTHANOX trial)	AC: Adequate Blinded: outcome assessors, data analysts ITT analysis Stopped early for insufficient accrual Sample size calculated a priori 100% follow-up	Enoxaparin 1.5 mg/kg daily × 3 months vs. Enoxaparin 1.5 mg/kg daily × 4 days followed by warfarin (target INR 2–3) × 3 months; 41% of time on target.	146 patients with cancer (solid or hematological; active or in remission but on treatment); with pulmonary embolism and/or DVT; minimum age of 18 years; minimum life expectancy of 3 months	Death, VTE, major bleeding at 3 months Death, minor bleeding, thrombocytopenia at 6 months	Funding: Aventis, Assistance Publique, Hospitaux de Paris
Cesarone 2003	Published only as abstract AC: Not clear Blinding: None, type of analysis not clear, Sample size calculation: not reported 97% follow-up.	Enoxaparin 100 UL/Kg twice daily × 3 months vs. coumadin (target INR 3) × 3 months.	199 patients with cancer with DVT	Death at 3 months	Funding: Not reported
Lee 2003 (CLOT trial)	AC: Adequate Blinded: outcome assessors, data analysts ITT analysis Sample size calculated a priori 99% follow-up	Dalteparin 200 IU/kg daily × 1 month followed by 150 IU/kg daily × 5 months vs. Dalteparin 200 IU/kg daily × 5–7 days followed by wafarin or acecumarol (target INR 2–3) × 6 months; 46% of time on target.	979 patients with active cancer and with DVT or pulmonary embolism or both; ECOG 1 or 2	Death, DVT, PE, VTE, major bleeding at 6 months Death at 1 year	Funding: Pharmacia
Dietcher 2006 (ONCENOX trial)	AC: Not clear Blinding: none ITT analysis Sample size not calculated a priori 89% follow-up	Enoxaparin 1 mg/kg twice daily × 5 days followed by 1–1.5 mg/kg daily × 175 days vs. Enoxaparin 1 mg/kg twice daily × 5 days followed by warfarin (target INR 2–3) for a total of 180 days	102 active patients with cancer with DVT and/or PE; minimum age of 18 years	Death, recurrent VTE, major bleeding, minor bleeding at 1 year	Funding: Aventis Pharmaceutical
Hull 2006 (LITE study)	AC: Adequate Blinded: outcome assessors, data analysts ITT analysis Sample size not calculated a priori 99% follow-up	Tinzaparin 175 antiXa/kg SQ daily for 12 weeks vs. UFH for 5 days followed by vitamin K antagonist (target INR 2–3) for 12 weeks.	200 patients with cancer (solid or hematological) with proximal DVT with or without PE; minimum age of 18 years; minimum life expectancy of 3 months	Death, recurrent VTE, major bleeding, minor bleeding, thrombocytopenia at 3 months Death, recurrent VTE at 1 year	Funding: Canadian Institute for Health Research, industry grant, Leo Pharmaceutical, Pharmion Pharmaceutical and Dupont Pharmaceutical.

* AC = allocation concealment; ITT = intention to treat analysis

† LMWH = Low molecular weight heparin; UFH = Unfractionated heparin

§ DVT = deep venous thrombosis; PE = pulmonary embolism; VTE = venous thromboembolism

### Methodological quality of included studies

The concealment of allocation was adequate in three trials [17,30,32] and unclear in the other 3 [16,29,31]. None of the studies blinded patients or caregivers, four studies blinded outcome assessors [17,30-32], and three studies blinded data analysts [17,30,32]. Five studies (all those published in full text) conducted ITT analysis [17,29-32]; this was not clear in the study published as an abstract [16]. Two studies reported a priori sample size calculations [30,32]. The percentage follow-up ranged from 89% to 100%. None of the studies was stopped early for benefit. The methodological quality varied by outcome. It was low for mortality, moderate for recurrent VTE, low for major bleeding and very low for minor bleeding, low for major bleeding and (Table 2). Table 2 also provides the absolute reductions in the risks of the different outcomes for a number of illustrative baseline risks, including low and high baseline risks.

**Table 2:** Summary of findings (SoF) table using GRADE methodology

**LMWH compared to VKA for patients with cancer requiring long term anticoagulation for VTE**						
**Patient or population**: patients with cancer requiring long term anticoagulation for VTE **Settings**: Outpatient **Intervention**: LMWH **Comparison**: VKA						

**Outcomes**	**Illustrative comparative ** risks* (95% CI)		**Relative** **effect** ** (95% CI)**	**No of ** **Participants** ** (studies)**	**Quality of** **the evidence ** ** (GRADE)**	**Comments**
					
	Assumed risk	Corresponding risk				

	**VKA**	**LMWH**				

**Mortality **(follow-up: 3–6 months)	**Population**		**RR 0.95 **(0.81 to 1.11)	1346 (4)	⊕⊕OO **low**^1,2^	
					
	**310 per 1000**	**294 per 1000 **(251 to 344)				
					
	**Low risk population**					
					
	**30 per 1000**	**28 per 1000 **(24 to 33)				
					
	**High risk population**					
					
	**1000 per 1000**	**950 per 1000 **(810 to 1110)				

**Recurrent VTE (binary) **(follow-up: 3–12 months)	**Population**		**RR 0.51 **(0.35 to 0.74)	1109 (4)	⊕⊕⊕O **moderate**^2^	
					
	**139 per 1000**	**71 per 1000 **(49 to 103)				
					
	**Low risk population**					
					
	**40 per 1000**	**20 per 1000 **(14 to 30)				
					
	**High risk population**					
					
	**160 per 1000**	**82 per 1000 **(56 to 118)				

**Major bleeding **(follow-up: 3–6 months)	**Low risk population**		**RR 1.05 **(0.53 to 2.1)	1120 (4)	⊕⊕OO **low**^2,3^	
					
	**30 per 1000**	**31 per 1000 **(16 to 63)				
					
	**High risk population**					
					
	**160 per 1000**	**168 per 1000 **(85 to 336)				

**Minor bleeding **(follow-up: 3–6 months)	**Low risk population**		**RR 0.85 **(0.53 to 1.35)	1120 (4)	⊕OOO **very low**^2,4^	
					
	**120 per 1000**	**102 per 1000 **(64 to 162)				
					
	**High risk population**					
					
	**500 per 1000**	**425 per 1000 **(265 to 675)				

*The basis for the **assumed risk **(e.g. the median control group risk across studies) is provided in footnotes. The **corresponding risk **(and its 95% confidence interval) is based on the assumed risk in the comparison group and the **relative effect **of the intervention (and its 95% CI).

CI: confidence interval; LMWH: low-molecular-weight heparin; RR: Risk ratio; VKA: vitamin K antagonists; VTE: venous thromboembolism

GRADE Working Group grades of evidence

**High quality**: Further research is very unlikely to change our confidence in the estimate of effect.

**Moderate quality**: Further research is likely to have an important impact on our confidence in the estimate of effect and may change the estimate.

**Low quality**: Further research is very likely to have an important impact on our confidence in the estimate of effect and is likely to change the estimate.

**Very low quality**: We are very uncertain about the estimate.

^1 ^RR = 0.95 and 95% CI = 0.81–1.11

^2 ^We could not obtain data for subgroups of patients with cancer in 11 RCTs

^3 ^RR = 1.05; 95% CI = 0.53–2.10

^4 ^Inconsistency was severe (I^2 ^= 65%)

### Effects of interventions

#### Survival

**Figure 3:**
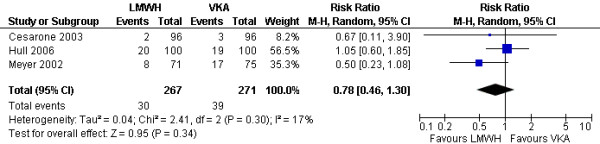
Comparison of the effects of LMWHs and vitamin K anatomists on survival (time to event analysis) in patients with cancer and venous thromboembolism.

We used time to event data reported by two studies [30,32] and supplied by the author of a third study [17]. The pooled analysis showed no statistically significant survival benefit of LMWH over VKA (HR = 0.96; 95% CI 0.81–1.14; I^2 ^= 0%) (Figure 3).

Three studies reported all cause mortality at three months [16,17,32]. The pooled analysis showed no statistically significant difference between LMWH and VKA (RR = 0.78; 95% CI 0.46–1.3; I^2 ^= 17%). In a sensitivity analysis excluding the study published as an abstract [16], the results remained non statistically significant (RR = 0.76; 95% CI = 0.37–1.58; I^2 ^= 58%). In a sensitivity analysis excluding the study that used a different initial anticoagulant in the two study arms [17], the results remained non statistically significant (RR = 0.52; 95% CI = 0.26–1.06; I^2 ^= 0%).

Three studies reported all cause mortality at 6 months [29,30,32]. The pooled analysis showed no statistically significant difference between LMWH and VKA (RR = 0.94; 95% CI = 0.79–1.11; I^2 ^= 0%).

**Figure 4:**
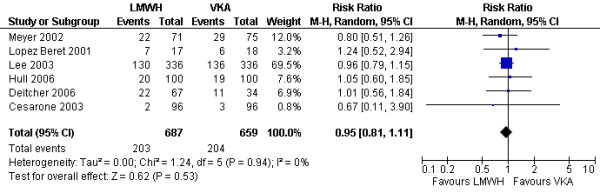
Comparison of the effects of LMWHs and vitamin K anatomists on mortality (categorical analysis) in patients with cancer and venous thromboembolism.

We finally pooled data from all studies irrespectively of the timing of outcome assessment and using the 6 months data from the study by Meyer et al. [32] The pooled analysis showed no statistically significant difference between LMWH and VKA (RR = 0.95; 95% CI = 0.81–1.11; I^2 ^= 0%) (Figure 4). In a sensitivity analysis excluding the study published as an abstract [16], the results remained non statistically significant (RR = 0.95; 95% CI = 0.82–1.12; I^2 ^= 0%). In a sensitivity analysis excluding the study that used a different initial anticoagulant in the two study arms [17], the results remained non statistically significant (RR = 0.94; 95% CI = 0.80–1.11; I^2 ^= 0%).

#### Recurrent venous thromboembolism

**Figure 5:**
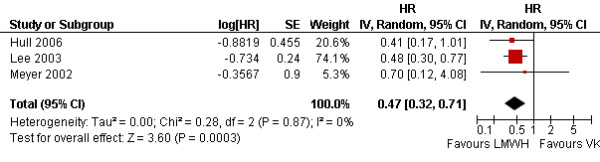
Comparison of the effects of LMWHs and vitamin K anatomists on recurrent venous thromboembolism (survival analysis) in patients with cancer and venous thromboembolism.

We used time to event data reported by two studies [30,32] and supplied by the author of a third study [17]. The pooled analysis showed a statistically significant benefit of LMWH over VKA (HR = 0.47; 95% CI = 0.32–0.71; I^2 ^= 0%) (Figure 5). Four studies reported binary data for venous thromboembolism [17,29,30,32]. The binary data analysis confirmed the results of the time to event analysis with a statistically significant benefit of LMWH over VKA (RR = 0.51; 95% CI = 0.35–0.74; I^2 ^= 0%). In a sensitivity analysis excluding the study that used a different initial anticoagulant in the two study arms [17], the results remained statistically significant (RR = 0.53; 95% CI = 0.35–0.80; I^2 ^= 0%). None of the studies reported DVT and pulmonary embolism as separate outcomes.

#### Bleeding outcomes

Four studies assessed bleeding outcomes [17,29,30,32]. The pooled analysis showed no statistically significant difference between LMWH and VKA for minor bleeding (RR = 0.85; 95% CI = 0.53–1.35; I^2 ^= 65%), major bleeding (RR = 1.05; 95% CI = 0.53–2.10; I^2 ^= 42%), and all bleeding (RR = 0.91; 95% CI = 0.64–1.31; I^2 ^= 50%). In a sensitivity analysis excluding the study that used a different initial anticoagulant in the two study arms [17], the results remained non statistically significant for all types of bleeding (minor bleeding: RR = 0.75; 95% CI = 0.41–1.39; I^2 ^= 73%; major bleeding: RR = 1.09; 95% CI = 0.39–3.08; I^2 ^= 61%; all bleeding: RR = 0.86; 95% CI = 0.53–1.38; I^2 ^= 62%).

#### Thrombocytopenia

Two studies assessed thrombocytopenia as an outcome [17,32]. The pooled analysis showed no statistically significant difference between LMWH and VKA (RR = 1.02; 95% CI = 0.60–1.74; I^2 ^= 0%). In a sensitivity analysis excluding the study that used a different initial anticoagulant in the two study arms [17], the results remained non statistically significant (RR = 0.94; 95% CI = 0.52–1.69).

None of the studies reported postphlebitic syndrome as an outcome.

## Discussion

For the long term treatment of venous thromboembolism in patients with cancer, LMWH compared to VKA provided no statistically significant survival benefit but a statistically and patient important reduction in venous thromboembolism. There was no statistically significant difference between LMWH and VKA in terms of bleeding outcomes or thrombocytopenia.

Our systematic approach to searching, study selection and data extraction should have minimized the likelihood of missing relevant studies. This increases the confidence in the internal validity of our findings. A major limitation of this review is our inability to include in the meta-analyses 11 eligible RCTs with subgroups of patients with cancer because relevant data was not reported and not obtainable from the authors. However, the inverted funnel plot for the outcome of all cause mortality did not suggest publication bias. This suggests that the treatment effect from those 11 RCTs should be similar to the one estimated from the included studies. One has to keep in mind that funnel plots have limited power to detect bias if the number of studies is small [10].

The pooled results for all cause mortality and bleeding outcomes showed moderate to severe heterogeneity. Unfortunately, the number of pooled studies was relatively small to explore the causes of heterogeneity by conducting subgroup analyses. However, the findings suggest that the trial that used a different initial anticoagulant in the two study arms is the source of heterogeneity [17].

Three published systematic reviews compared LMWH and VKA in the long treatment of venous thromboembolism [7-9]. Two of these systematic reviews showed no statistically significant reduction of recurrent venous thromboembolism by LMWH compared to VKA when the meta-analysis is not restricted to patients with cancer [7,9]. However, our meta-analysis shows a significant reduction in recurrent venous thromboembolism in patients with cancer. The reason for this differential effect in patients with cancer is not clear. A similar differential effect of anticoagulants has been found in the initial treatment of venous thromboembolism where LMWH was superior to UFH in patients with cancer but not in patients without cancer [36].

Of the three published systematic reviews comparing LMWH and VKA in the long treatment of venous thromboembolism [7-9], only the study by Ioro et al. conducted a meta-analysis in the subgroup of patients with cancer and found no statistically significant difference in mortality (OR = 1.13; 95% CI 0.54, 2.38). This finding is consistent with the results of our meta-analysis. While the reduction in venous thromboembolic events with LMWH in patients with cancer is expected to reduce thrombosis related mortality, this did not translate into a reduction in all cause mortality. This finding is not apparently explained by an increase in any specific-cause mortality (e.g. fatal bleeding), but might be due to the lack of power to detect a reduction in all cause mortality especially that the results suggest a trend in that direction.

We were not able to conduct subgroup analyses based on type of cancer because of the lack of data. Such analyses would be interesting because of the survival benefits of LMWH in patients with limited small cell lung cancer [37] and of VKA in patients with small cell lung cancer [38] that are independent of any antithrombotic effects.

## Conclusion

The decision for a patient with cancer and venous thromboembolism to start long term LMWH versus oral anticoagulation should balance the benefits and downsides and integrate the patient's values and preferences for outcomes and management options [39]. While LMWH decreases the incidence of venous thromboembolism and possibly of death, we speculate that it might be more costly and less acceptable because of its subcutaneous route of administration.

Future research should compare LMWH to other anticoagulants such as ximelagatran and fondaparinux. There is also a need for research assessing patients' values and preferences regarding long term anticoagulant agents for treating venous thromboembolism. Researchers should consider making the raw data of RCTs available for individual patient data meta-analysis. Further RCTs including subgroups of patients with cancer should report separate results for these subgroups.

## List of abbreviations

CI: Confidence interval; DVT: Deep vein thrombosis; GRADE: Grading of Recommendations Assessment Development and Evaluation; HR: Hazard ratio; ITT: Intention-to-treat; LMWH: Low-molecular-weight-heparin; OR: Odds ratio; RCT: Randomized clinical trial; RR: Relative risk; SCLC: Small cell lung cancer; UFH: Unfractionated heparin; VKA: Vitamin K antagonist; VTE: Venous thromboembolism.

## Competing interests

Schünemann: no personal payments from for-profit sponsors, but he received research grants and honoraria that were deposited into research accounts or received by a research group that he belongs to from AstraZeneca, Amgen, Chiesi Foundation, Lily, and Pfizer, Roche and UnitedBioSource for development or consulting regarding quality of life instruments for chronic respiratory diseases and as lecture fees related to the methodology of evidence based practice guideline development and research methodology. Institutions or organizations that he is affiliated with likely receive funding from for-profit sponsors that are supporting infrastructure and research that may serve his work.

## Authors' contributions

EAA: protocol development, search for trials, screening, data extraction, data analysis, manuscript drafting, review coordination. MB: screening, data extraction. SR: screening, data extraction. IT: screening, data extraction. FS: screening, data extraction. PM: data analysis, methodological advice. HJS: protocol development, search for trials, data extraction, data analysis, methodological advice. All authors read and approved the final manuscript.

## Supplementary Material

Additional file 1 "Search strategies used for the electronic databases; parenteral anticoagulation to prolong survival systematic review". search strategy. Click here for file
